# Validated Colorimetric Assay of Clonidine Hydrochloride from Pharmaceutical Preparations

**Published:** 2016

**Authors:** Andreia Corciova

**Affiliations:** *Department of Drugs Analysis, “Grigore T. Popa” University of Medicine and Pharmacy, Faculty of Pharmacy, Iasi, Romania.*

**Keywords:** Clonidine hydrochloride, Quantitative assay, UV-Vis spectrophotometry, Ion-pair complexes, Validation

## Abstract

Clonidine hydrochloride is an antihypertensive agent used for migraine prophylaxis, attention deficit hyperactivity disorder, menopausal flushing and Tourette syndrome. The quantity of the active substance in pharmaceutical preparations must be within specific limits, in agreement with the respective label claim. Therefore, the aim of this study was to establish the conditions for two spectrophotometric methods for clonidine determination, based on the formation of the ion pair complex between clonidine hydrochloride and thymol blue/bromophenol blue. A Jasco UV-Vis 530 spectrophotometer was used for the analysis and the maxim absorbance was measured at 418 nm/448 nm against blank solution. After validation, the methods were used for quantification of clonidine hydrochloride in two commercial samples (tablets). The recovery of active substance varies between 98.06 and 100.13 % without interferences from the excipients.

## Introduction

**Figure 1:**
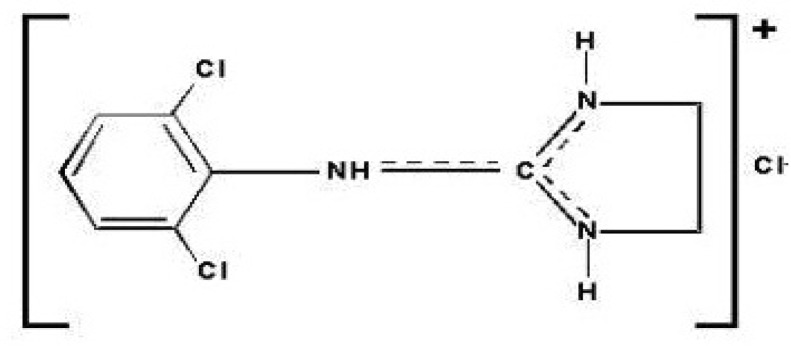
Chemical structure of clonidine hydrochloride

Clonidine (Figure 1),* N*-(2, 6-dichlorophenyl)-4, 5-dihydro-1*H*-imidazol-2-amine, is an imidazolinic derivative and exists as a mesomeric compound.

The substance is an α_2_-adrenergic agonist. The decrease of blood pressure is mainly due to a central action, exerted on the reticular nucleus of the bulb, with consequent reduction of peripheral sympathetic and vagal cardio-inhibitory reflexes. As a result of the reduced peripheral sympathetic function, the levels of norepinephrine in plasma decrease. It also acts upon specific receptors for imidazoline structure - I_1_ receptors that mediate the sympatho-inhibitory actions of imidazolines to lower the blood pressure.

Efficiency is highest 2-3 weeks after starting the treatment. It is used in hypertension - moderate and severe forms (commonly associated with diuretics, possibly with other antihypertensive), for prophylaxis of migraine and other forms of recurrent vascular headache and to treat menopausal flushing (1, 2). In an extended release formulation, FDA approved it for treatment of attention-deficit hyperactivity disorder (ADHD), from 2010 (3-5). Also, clonidine is used in Tourette syndrome (especially for tics) (6, 7). 

It is contraindicated in patients with a history of depressive psychosis. It must be avoided or used with caution during pregnancy, severe cerebral arteriosclerosis, Raynaud's disease and obliterative thromboangiitis. Clonidine is contraindicated for drivers and those with other professions that require intense concentration and prolonged mental functions. It must not be administrated in association with alcohol and central depressants (barbiturates and other sedatives). Clonidine may present the next side effects: frequent dry mouth, constipation, drowsiness and sedation, relatively rare nausea, anorexia, sinus bradycardia, orthostatic hypotension, fluid retention (at baseline). Abrupt withdrawal may cause marked hypertension (rebound effect), headache, nervousness, tremor, gastrointestinal disorders (1, 2).

Clonidine hydrochloride is soluble in water and in anhydrous ethanol. The officialized analysis methods for clonidine hydrochloride are: a potentiometric titrimetric assay for drug determination according to European Pharmacopoeia and British Pharmacopoeia, and a spectrophotometric method based on the reaction with bromothymol blue for clonidine quantification in tablets, as described in British Pharmacopoeia (8, 9). The literature presents several methods for determination of clonidine hydrochloride from tablets, most of them being high performance liquid chromatography (HPLC) (10-13). There are a few colorimetric methods using reactions with sodium nitroprusside in presence of sodium hydroxide or with bromocresol green and supracen violet (14-17). Other methods used for clonidine hydrochloride analysis from tables are capillary electrophoresis (18), potentiometric assay (19), spectrophotometric titration in different non-aqueous solvents (20), derivative ratio spectrophotometry method and solvent extraction flow injection analysis using bromophenol blue (21).

Ion pair extraction has received considerable interest for quantitative determination of drugs because is a simple, cheap, sensitive and rapid method unlike other methods that are modern, expedient but involve complex instruments which may not be available in most laboratories. The aim of this study was to describe two methods for clonidine hydrochloride assay, based on the ion pair extraction technique, using thymol blue and bromophenol blue (22). In order to validate the proposed methods, we investigated the following validation parameters: linearity, limit of detection, limit of quantification, precision and accuracy in accordance with the bioanalytical method validation guidelines (23-26).

## Experimental

*Chemicals and reagents*

- Clonidine hydrochloride pure reference substance was supplied by Sigma Aldrich.
- Clonidine hydrochloride tablets containing 0.15 mg drug were purchased from the pharmacy (two commercial products).
- Bromophenol blue solution was prepared by dissolving 0.1 g bromophenol blue (Riedel de Haen) in 20 mL methanol and adding distilled water to 100 mL.
- Thymol blue solution was prepared by dissolving 0.1 g thymol blue (Riedel de Haen) in 100 mL methanol.
- Acetate buffer solution pH = 3 was prepared by dissolving 12 g sodium acetate in 50 mL distilled water, then adding 6 ml acetic acid and completing to 100 mL with distilled water.
- Methanol p.a, Chloroform p.a., Acetic acid p.a., Sodium acetate p.a.(Chemical Company)

*Methods used for ion-pair formation* ***:***

- Method using bromophenol blue: 1 mL from each working solution was mixed into a separating funnel with 1 mL acetate buffer solution of pH = 3 and 1 mL of bromophenol blue solution. The complex was extracted for 5 min with two portions of 5 mL chloroform, passed through anhydrous sodium sulphate and completed with chloroform. After 30 min, the absorbance was measured at the wavelength of maximum absorption 448 nm, versus a blank solution prepared in similar conditions.
- Method using thymol blue: 1 mL from each working solution was mixed into a separating funnel with 1 mL acetate buffer solution of pH = 3 and 2 mL of thymol blue solution. The complex was extracted for 5 min with two portion of 5 mL chloroform, passed through anhydrous sodium sulphate and completed with chloroform. After 20 min, the absorbance was measured at the wavelength of maximum absorption-418 nm, versus a blank solution prepared in similar conditions.

*Preparation of stock standard solution* *100* *μg/mL:*

10 mg of clonidine hydrochloride was dissolved in 3 mL methanol and completed to 100 mL with distilled water.

*Working solutions*

Containing from 0.5-7.5 μg/mL clonidine hydrochloride were obtained by diluting the stock solution with distilled water.

*Preparation of sample solutions*:

 Twenty tablets from each sample product were weighed. Their average weight was calculated and then they were finely powdered in a glass mortar. Two quantities of powder, each equivalent to 30 mg clonidine hydrochloride, were weighed and transferred into a 100 mL volumetric flask. 10 mL methanol and 20 mL distilled water were added to both flasks and stirred for 10 min to dissolve the drug. The solutions were filtered through Whatman filter paper and completed to 100 mL with distilled water. The sample solutions were marked as Sample 1 and Sample 2. The procedure was continued as described on ion-pair formation procedure.

*Instrument*

A Jasco V 530 double beam UV-Vis spectrophotometer was used. All the measurements were made in 1.0 cm quartz cells at a scan speed of 1000 nm min^-1^ and scan range of 400-600 nm, fixed slit width of 2 nm. 

## Results and Discussion

*Optimization of variables*

- Selection of solvents for extraction of the complex

1 mL of working solution was mixed with 1 mL acetate buffer solution of pH = 3 and 1 mL of bromophenol blue solution/2 mL thymol blue solution. Then the complex was extracted with CCl_4_, chloroform and dichloromethane and the absorbances were measured at 448 nm/418 nm. 

Because the maximum absorbance was obtained with chloroform (Table 1), it was chosen as extraction solvent. 

**Table 1:** Selection of solvents for extraction of the complex

**Solvents**	**Absorbance ± SD**	
	**Bromophenol blue **	**Thymol blue**
CCl_4_	-	-
Chloroform	1. 0896 ± 0.003	0.8796 ± 0.007
Dichloromethane	0.4315 ± 0.036	0.2673 ± 0.005

- Selection of pH and reaction time

**Figure 2:**
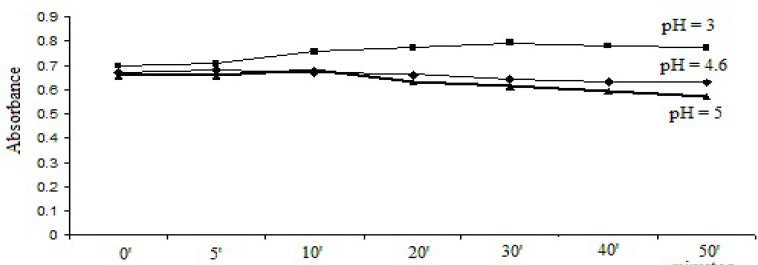
Selection of pH dependence and reaction time for method with bromophenol blue solution.

**Figure 3:**
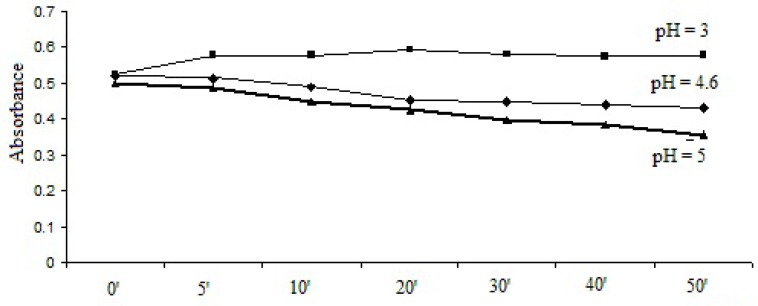
Selection of pH dependence and reaction time for method with thymol blue solution.

1 mL bromophenol blue solution/2 mL thymol blue solution was added to the 1 mL working solution and the pH was adjusted to pH = 3, pH = 4.6 and pH = 5. After extraction, the absorbances were recorded at different time intervals. The results are represented in Figure 2 and Figure 3.

As it can be seen, the optimal stabilities of the formed complexes were obtained at pH = 3 with both dyes and the optimum reaction time was 30 min for bromophenol blue solution method and 20 min for thymol blue solution method.

- Selection of dye concentration and quantity

The influence of the concentration of dyes and the quantity used are presented in Table 2 and Table 3. 

**Table 2:** Selection of dye concentration

**Dyes**	**0.05%**	**0.1%**	**0.5%**
Bromophenol blue solution	0.1955	**0.6911**	0.5425
Thymol blue solution	0.4825	**0.5862**	0.3602

**Table 3:** Selection of dye quantity

**Dyes**	**0.5 mL**	**1 mL**	**1.5 mL**	**2 mL**	**2.5 mL**
Bromophenol blue solution	0.2132	**0.6763**	0.6752	0.6743	0.6729
Thymol blue solution	-	0.5862	0.7108	**0.8856**	0.8662

The optimum concentration of the two dyes used was 0.1% and the quantity of dye was established at 1 mL for bromophenol blue solution and 2 mL for thymol blue solution. 

*Validation of methods*

- Linearity

**Figure 4:**
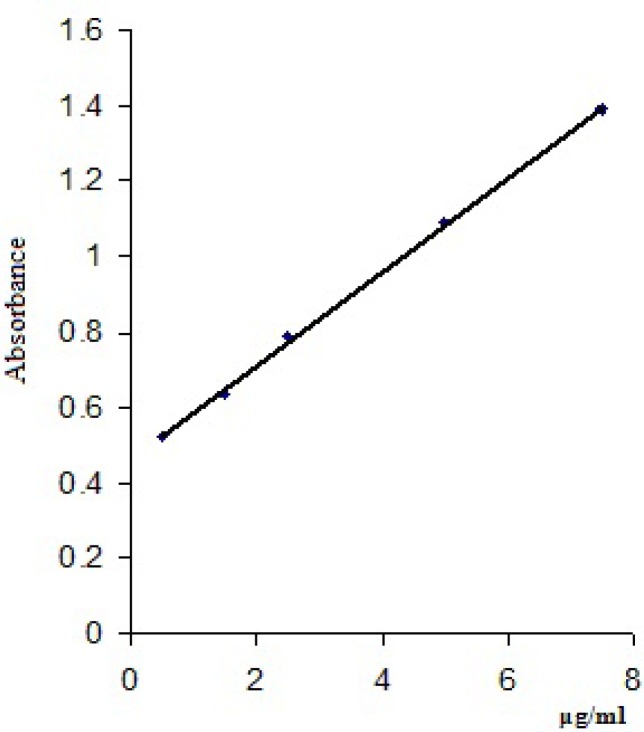
Calibration curve for clonidine hydrochloride with bromophenol blue solution

**Figure 5:**
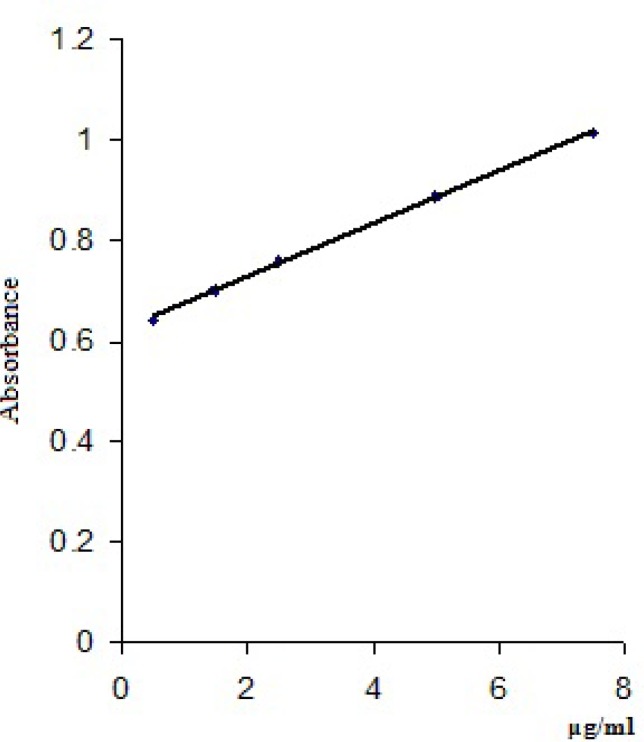
Calibration curve for clonidine hydrochloride with thymol blue solution

Three determinations for each concentration were made and a mean value of the absorbances read at 448 nm/418 nm was calculated (Table 4). The calibration curves were obtained by plotting the mean values of the absorbances of clonidine hydrochloride *vs* clonidine hydrochloride concentrations (μg/mL) and are presented in Figure 4 and Figure 5. 

**Table 4:** Absorbance values for linearity study

**Statistical data**	**Method with bromophenol blue solution**	**Method with thymol blue solution**
Pearson Coefficient (r^2^)	0.999	0.9995
Standard Error	0.012978	0.003889
Intercept	0.4628	0.6213
Slope	0.1245	0.0528
Limit of detection	0.039115	0.011721
Limit of quantification	0.11853	0.035519

Statistical data regarding clonidine hydrochloride determination are shown in Table 5.

**Table 5:** Statistical data regarding clonidine hydrochloride determination

**Statistical data**	**Method with bromophenol blue solution**	**Method with thymol blue solution**
Pearson Coefficient (r^2^)	0.999	0.9995
Standard Error	0.012978	0.003889
Intercept	0.4628	0.6213
Slope	0.1245	0.0528
Limit of detection	0.039115	0.011721
Limit of quantification	0.11853	0.035519

- Repeatability (System precision)

For determination of repeatability, solutions at concentrations of 1.5 μg/mL in six replicates were used. The absorbances were measured and then the average, SD and % RSD were calculated. Table 6 shows the experimental data of the absorbances related to the precision of the system. 

**Table 6:** Experimental data of the absorbances relating to the precision of the system

**No**	**Absorbance**	
	**Method with bromophenol ** **blue solution**	**Method with thymol blue solution**
1.	0.6759	0.7028
2.	0.6692	0.6999
3.	0.6649	0.6983
4.	0.6703	0.7058
5.	0.6732	0.711
6.	0.6697	0.7037
Average	0.6705	0.7035
SD	0.0037	0.0045
% RSD	0.5587	0.6420

Relative standard deviation (RSD) was found to be 0.5587% and 0.6420% respectively, which is lower than the maximum 2% proposed by the European standards, so the system is considered to be precise.

- Method precision and accuracy

To investigate the method precision, solutions of three concentration levels were chosen and three determinations were made for each of them. Tables 7 and 8 present the calculated concentrations and the recovery of the methods in the same day, at different times (8.00 a.m., 12.00 a.m. and 16.00 p.m.) and in 3 consecutive days (day 1, 2 and 3). 

**Table 7:** Calculated concentrations and the recovery of the method in the same day

**Method with bromophenol blue solution**								
**8.00 a.m.**			**12.00 a.m.**			**16.00 a.m.**		
**Abs**	**Calc. ** **conc.**	**% Recovery**	**Abs**	**Calc. ** **conc.**	**% Recovery**	**Abs**	**Calc. ** **conc.**	**% Recovery**
0.6495	1.4995	99.97	0.6499	1.5028	100.18	0.6489	1.4947	99.65
0.6488	1.4939	99.59	0.6478	1.4859	99.06	0.6478	1.4859	99.06
0.6477	1.4851	99.00	0.65	1.5036	100.24	0.648	1.4875	99.17
0.7745	2.5036	100.14	0.7737	2.4971	99.88	0.7746	2.5044	100.17
0.7721	2.4843	99.37	0.7742	2.5012	100.04	0.7738	2.4979	99.91
0.7739	2.4987	99.95	0.7737	2.4971	99.88	0.7741	2.5004	100.01
1.0852	4.9991	99.98	1.0802	4.9590	99.18	1.0803	4.9598	99.19
1.0829	4.9807	99.61	1.0805	4.9614	99.22	1.081	4.9654	99.31
1.0859	5.0048	100.09	1.0811	4.9662	99.32	1.0839	4.9887	99.77
Average		99.75	Average		99.67	Average		99.58
SD		0.379	SD		0.468	SD		0.412
% RSD		0.380	% RSD		0.469	% RSD		0.414
**Method with thymol blue solution**								
**8.00 a.m.**			**12.00 a.m.**			**16.00 a.m.**		
**Abs**	**Calc. ** **conc.**	**% Recovery**	**Abs**	**Calc. ** **conc.**	**% Recovery**	**Abs**	**Calc. ** **conc.**	**% Recovery**
0.6998	1.4867	99.11	0.701	1.5094	100.63	0.6999	1.4886	99.24
0.7001	1.4924	99.49	0.7009	1.5075	100.50	0.7012	1.5132	100.88
0.7011	1.5113	100.75	0.7009	1.5075	100.50	0.7	1.4905	99.36
0.7522	2.4791	99.16	0.7537	2.5075	100.30	0.7525	2.4848	99.39
0.754	2.5132	100.53	0.7529	2.4924	99.69	0.752	2.4753	99.01
0.7538	2.5094	100.37	0.7528	2.4905	99.62	0.7522	2.4791	99.16
0.8849	4.9924	99.84	0.8845	4.9848	99.69	0.8839	4.9734	99.46
0.8852	4.9981	99.96	0.8839	4.9734	99.46	0.8839	4.9734	99.46
0.8851	4.9962	99.92	0.8828	4.9526	99.05	0.886	5.0132	100.26
Average		99.90	Average		99.94	Average		99.58
SD		0.579	SD		0.556	SD		0.59
% RSD		0.580	% RSD		0.556	% RSD		0.60

**Table 8:** Recovery values of the method in three consecutive days

		% RSD		% RSD		% RSD
Method with bromophenol blue solution	Day 1	0.42	Day 2	0.56	Day 3	0.61
Method with thymol blue solution	Day 1	0.57	Day 2	0.65	Day 3	0.59

The mean recovery of the tests made in the same day for the method with bromophenol blue solution was in the range 99,00-100,24%, with SD = 0.379-0.468 and % RSD = 0.38-0.469 and for the method with thymol blue solution was in the range 99.01-100.88%, with SD = 0.556-0.59 and % RSD = 0.556-0.60.

The RSD values in 3 different days were in the range 0.42-0.61 for the bromophenol blue solution method and in the range 0.57-0.65 for thymol blue solution method. 

As it can be seen the RSD values are lower than maximum 5% proposed by the European standards, therefore the method is accurate.

*Application on tablets*

The applicability of the two methods was demonstrated on two samples from pharmacy (Sample 1 and Sample 2), in 3 consecutive days, on 3 replicates each day. Table 9 presents the average of the 3 replicates of mg clonidine hydrochloride/tablet found and % recovery, considering that the declared concentration is 0.15 mg/tablet. 

**Table 9:** Clonidine hydrochloride concentrations found in samples

	**Method with bromophenol blue solution**			
	**Sample 1**		**Sample 2**	
	**mg found** **/tablet ** **± SD**	**%** **Recovery**	**mg found** **/tablet ** **± SD**	**%** **Recovery**
Day 1	0.1490 ± 0.003	99.33	0.1471 ± 0.031	98.06
Day 2	0.1499 ± 0.012	99.93	0.1475 ± 0.007	98.33
Day 3	0.1502 ± 0.029	100.13	0.1486 ± 0.005	99.06
	**Method with thymol blue solution**			
	**Sample 1**		**Sample 2**	
	**mg found** **/tablet ** **± SD**	**%** **Recovery**	**mg found** **/tablet ** **± SD**	**%** **Recovery**
Day 1	0.1495 ± 0.026	99.66	0.1472 ± 0.008	98.13
Day 2	0.1503 ± 0.015	100.2	0.1477 ± 0.006	98.46
Day 3	0.1492 ± 0.079	99.46	0.1478 ± 0.017	98.53

The results obtained in the 3 days comply with the limits imposed by the regulations of Romanian Pharmacopoeia 10^th^ edition, Monography - Compressi [± 10%] (27) and the recoveries of active substance are in agreement with their respective label claim. Also, the results were compared with those obtained by the spectrophotometric method stipulated in European Pharmacopoeia and the differences were insignificant. 

## Conclusion

This paper establishes the conditions for clonidine hydrochloride assay by two spectrophotometric methods using bromophenol blue solution and thymol blue solution in acid medium. The methods are simple, cheap, accurate and precise and the results obtained showed that the proposed method for quantification of clonidine hydrochloride comply with validation parameters and can be applied successfully to the determination of substance in marketed formulations. 
